# Prognostic value of baseline hemoglobin‐to‐red blood cell distribution width ratio in small cell lung cancer: A retrospective analysis

**DOI:** 10.1111/1759-7714.13330

**Published:** 2020-02-22

**Authors:** Fangfang Wu, Shaoxing Yang, Xiuhua Tang, Wenjing Liu, Haoran Chen, Hongjun Gao

**Affiliations:** ^1^ PLA 307 Clinical College Anhui Medical University Beijing China; ^2^ Department of Pulmonary Oncology The Fifth Medical Center of Chinese PLA General Hospital Beijing China

**Keywords:** Hemoglobin, prognosis, red blood cell distribution width, small cell lung cancer

## Abstract

**Background:**

This study aimed to investigate the prognostic value of baseline hemoglobin‐to‐red blood cell distribution width ratio (HRR) in patients with small cell lung cancer (SCLC).

**Methods:**

We retrospectively analyzed the medical records of patients with newly diagnosed SCLC who had received first‐line chemotherapy at the Department of Pulmonary Oncology of the PLA 307 Hospital between January 2008 and October 2018. The optimal cutoff value of the continuous variables was determined using the X‐tile software. Univariate and multivariate analyses were conducted using Cox proportional hazard models. The Kaplan‐Meier method was used for survival analysis, with differences tested using the log‐rank test.

**Results:**

A total of 146 patients were included. The cutoff value for HRR was determined as 0.985. Statistically significant differences were observed in sex, smoking history, stage, radiotherapy combination, neutrophil‐to‐lymphocyte ratio, platelet‐to‐lymphocyte ratio, hemoglobin, and red blood cell distribution width between the high and low HRR groups. The median overall survival (OS) was nine and 17.5 months in the low and high HRR groups, respectively (*P* < 0.001). The median progression‐free survival (PFS) was five and 8.5 months, respectively (*P* < 0.001). Univariate and multivariate analyses showed low HRR to be an independent predictor of a poor prognosis for OS (hazard ratio = 3.782; 95% confidence interval, 2.151–6.652; *P* < 0.001) and PFS (hazard ratio = 2.112; 95% confidence interval, 1.195–3.733; *P* = 0.01) in SCLC.

**Conclusion:**

Low HRR was associated with poorer OS and PFS in patients with SCLC and can be a potentially valuable prognostic factor for these patients.

**Key points:**

The prognostic value of the baseline hemoglobin‐to‐red blood cell distribution width ratio was evaluated in patients with small cell lung cancer.

In this population, this ratio was an independent predictor of overall survival and progression‐free survival. This ratio, an inexpensive and routine parameter, can be used as a prognostic factor in small cell lung cancer.

## Introduction

Small cell lung cancer (SCLC) is a highly invasive neuroendocrine tumor accounting for about 15%–20% of lung cancer cases.^1^ The prognosis is poor because of the rapid multiplication rate, high degree of malignancy, and tendency for distant metastases. Although SCLC is sensitive to chemoradiotherapy in the early stages, most patients will develop tumor resistance or recurrence within one year of treatment,^2^ which severely limits the overall survival (OS) of patients. Inrecent years, numerous studies have examined the prognosis of SCLC. The Glasgow prognostic score,^3^ alkaline phosphatase level,^4^ and lactate dehydrogenase level,^5^ among other factors, have shown prognostic value, but no relatively uniform cutoff value for these factors has been established. Some molecular markers, such as exonic circular RNAs,^6^ 3‐microRNAs,^7^ circulating tumor cells,^8^ and serum P53 antibody,^9^ have been confirmed to be associated with survival in patients with SCLC; however, in clinical practice, the usefulness of these molecular markers is limited by their complex and expensive detection methods. Therefore, identifying more economical, convenient, and effective biomarkers to evaluate the prognosis of patients with SCLC is of great significance.

Complete blood count (CBC) is a routine test for patients with tumors on admission. A number of studies have shown that the platelet count,^5^ neutrophil‐to‐lymphocyte ratio (NLR), platelet‐to‐lymphocyte ratio (PLR),^10^ and other CBC‐derived indicators are correlated with the prognosis of patients with tumors. Hemoglobin (Hb), as an important CBC parameter, reflects the degree of anemia in patients. Studies have shown that the Hb level can be an independent predictor of prognosis in patients with colorectal cancer,^11^ gastric cancer,^12^ liver cancer,^13^ endometrial cancer,^14^ SCLC,^5^ and other cancers.

Red blood cell distribution width (RDW) is another important CBC parameter. RDW has been used for the diagnosis and differential diagnosis of various types of anemia.^15^ In recent years, changes in RDW have been found to be related to the inflammatory state of the body,^16^, ^17^ which has been shown to affect the development of tumors.^18^, ^19^ Therefore, researchers explored the relationship between RDW and the prognosis of some malignant tumors and confirmed that a high RDW is associated with a poor prognosis, such as in breast cancer,^20^ esophageal cancer,^21^ non‐small cell lung cancer (NSCLC),^22^ SCLC,^23^ and other tumors.

Based on research related to Hb and RDW in malignant tumors, Sun *et al*.^24^ was the first to report the prognostic value of the baseline Hb‐to‐RDW ratio (HRR) in esophageal squamous cell carcinoma (ESCC) in 2016. They revealed that HRR was an important prognostic indicator of ESCC, and similar results have also been reported for other types of cancers^25^, ^26^; however, to date, no studies have evaluated the clinical significance of HRR in the prognosis of SCLC. Therefore, this study aimed to assess the prognostic role of baseline HRR in SCLC and to further explore the potential relationship between HRR and the clinical features of patients with SCLC.

## Methods

### Patients

Patients with newly diagnosed SCLC who had received first‐line chemotherapy at the Department of Pulmonary Oncology of the People's Liberation Army (PLA) 307 Hospital between January 2008 and October 2018 were enrolled into the study. The inclusion criteria for the study were as follows: (i) SCLC diagnosed by histopathological analysis; (ii) adequate imaging data, such as CT and MRI data, for tumor staging; (iii) no previous antitumor treatment, including radiotherapy, chemotherapy, immunotherapy, and targeted treatment; (iv) an Eastern Cooperative Oncology Group (ECOG) score of 0–1; (v) complete blood test results of the hospital‐based laboratory to establish HRR, determined within one week before chemotherapy; (vi) routine blood and blood biochemistry findings that met the requirements for chemotherapy; and (vii) signed informed consent for chemotherapy and follow‐up. The exclusion criteria for the study were as follows: (i) the presence of other types of tumors; (ii) the presence of additional systemic hematological or immunological diseases; (iii) Hb < 9 g/dL; (iv) previous long‐term hormone therapy; (v) less than two cycles of first‐line continuous chemotherapy; and (vi) infections or other inflammatory diseases before blood sample collection. This study was approved by the Ethics Committee of PLA 307 Hospital. As this was a retrospective study, the ethics committee waived the requirement for informed consent.

### Clinical data collection

Clinical data, including age, sex, smoking history, drinking history, staging, first‐line chemotherapy regimens, and first‐line two cycle efficacy evaluation results, were recorded at the time of diagnosis. Smoking history was defined as continuous or cumulative smoking for more than six months. Drinking history was defined as long‐term regular drinking of up to 20 g/day (140 g/week) and up to 10 g/day (70 g/week) in men and women, respectively.^27^ The baseline HRR was calculated using the CBC values. The HRR value was calculated as Hb (g/dL) divided by RDW (%).

### Tumor evaluation criteria

Tumor staging was based on the eighth edition of the staging criteria issued by the International Association for the Study of Lung Cancer.^28^ The efficacy evaluations included complete response (CR), partial response (PR), stable disease (SD), and progressive disease (PD), according to the response evaluation criteria in solid tumors (RECIST), version 1.1.

### Observation indicators

The following observation indicators were used: HRR, efficacy evaluation for the second cycle, OS, and progression‐free survival (PFS). OS was defined as the time from initial treatment to death or the last follow‐up, whereas PFS was defined as the time from the onset of first‐line chemotherapy to the date of disease progression or death.

### Follow‐up

The follow‐up, consisting of regular visits to the hospital or telephone assessments, began in February 2008 and ended on 31 July 2019. The follow‐up rate was 100.0%; the shortest follow‐up time was five months, and the longest was 138 months.

### Statistical analysis

SPSS 22.0 (IBM Corp., Chicago, IL, USA) software was used for all statistical analyses. Clinicopathological features were compared between groups using the Chi‐square test or Fisher's exact test. The best truncation value of the continuous variables was obtained using X‐tile software^29^, ^30^ (version 3.6.1, Yale University, USA). The Kaplan–Meier method was used for survival analysis, and the log‐rank test was used to evaluate differences in survival. In univariate and multivariate analyses, the Cox proportional risk regression model (backward selection) was used to evaluate the independent risk factors for OS and PFS. All statistical tests were bilateral probability tests with α = 0.05, and *P* < 0.05 was considered statistically significant.

## Results

### Patient characteristics and treatments

A total of 146 patients (114 men and 32 women) were included in this study. The basic clinical and pathological characteristics of the patients are shown in Table 1. The mean age at disease onset was 57 years (range, 19–74 years). Smoking and drinking histories were noted in 74% and 41.8% of the patients, respectively. In the study population, 87 and 59 patients had extensive disease (ED) and limited disease (LD), respectively. The most commonly used first‐line chemotherapy regimen was etoposide plus lobaplatin (49.3%), followed by etoposide combined with cisplatin (41.1%) and etoposide combined with carboplatin (9.6%). A total of 48 patients did not receive radiotherapy, 66 patients received sequential radiotherapy, and 32 patients received synchronous radiotherapy during first‐line chemotherapy. No patients were evaluated as CR after first‐line chemotherapy. Among all patients, 82.9% showed PR after two cycles of chemotherapy. The NLR was between one and 19, with a mean of three and a median of two. The PLR ranged between 52 and 757, and the mean and median were 167 and 149, respectively. The median concentration of Hb in the entire patient population was 13.7 g/dL (range, 8.1–17.1 g/dL), and 21 patients were anemic (female and male patients with Hb <11 and Hb <12 g/dL, respectively). The median RDW value was 12.8% (range, 11.3%–21.8%). The median and mean of HRR were both one. The X‐tile software determined the optimal cutoff values as 0.985 for HRR (both OS and PFS), 10.9 g/dL (OS) and 12.3 g/dL (PFS) for Hb, 14.1% (OS) and 13.6% (PFS) for RDW, three (both OS and PFS) for NLR, and 165 (both OS and PFS) for PLR.

**Table 1:** Clinical features and characteristics of the study population stratified by the Hb/RDW ratio

			Hb/RDW ratio				
Variable	Total	%	<0.985	%	≥0.985	%	*P*‐value
Sex							
Female	32	21.9	15	41.7	17	15.5	0.001
Male	114	78.1	21	58.3	93	84.5	
Age							
≤60	86	58.9	21	58.3	65	59.1	0.936
>60	60	41.1	15	41.7	45	40.9	
Smoking history							
No	38	26	17	47.2	21	19.1	0.001
Yes	108	74	19	52.8	89	80.9	
Alcohol history							
No	85	58.2	23	63.9	62	56.4	0.427
Yes	61	41.8	13	36.1	48	43.6	
Stage							
LD	59	40.4	9	25	50	45.5	0.03
ED	87	59.6	27	75	60	54.5	
First‐line chemotherapy							
Etoposide + cisplatin	60	41.1	13	36.1	47	42.7	0.771
Etoposide + lobaplatin	72	49.3	19	52.8	53	48.2	
Etoposide + carboplatin	14	9.6	4	11.1	10	9.1	
Combination with radiotherapy							
No	48	32.9	18	50	30	27.3	0.036
Sequential radiotherapy	66	45.2	11	30.6	55	50	
Synchronous radiotherapy	32	21.9	7	19.4	25	22.7	
Two‐cycle efficacy evaluation							
PR	121	82.9	27	75	94	85.5	0.329
SD	17	11.6	6	16.7	11	10	
PD	8	5.5	3	8.3	5	4.5	
NLR							
≤3	97	66.4	14	38.9	83	75.5	<0.001
>3	49	33.6	22	61.1	27	24.5	
PLR							
≤165	92	63	12	33.3	80	72.7	<0.001
>165	54	37	24	66.7	30	27.3	
Hb (g/dL)							
≤10.9	17	11.6	17	47.2	0	0	<0.001
>10.9	129	88.4	19	52.8	110	100	
RDW (%)							
≤14.1	131	89.7	22	61.1	109	99.1	<0.001
>14.1	15	10.3	14	38.9	1	0.9	

*P*‐values <0.05 are considered statistically significant.

ED, extensive disease; Hb, hemoglobin; LD, limited disease; NLR, neutrophil/lymphocyte ratio; PD, progressive disease; PLR, platelet/lymphocyte ratio; PR, partial response; RDW, red blood cell distribution width; SD, stable disease.

### Survival analysis

**Figure 1:**
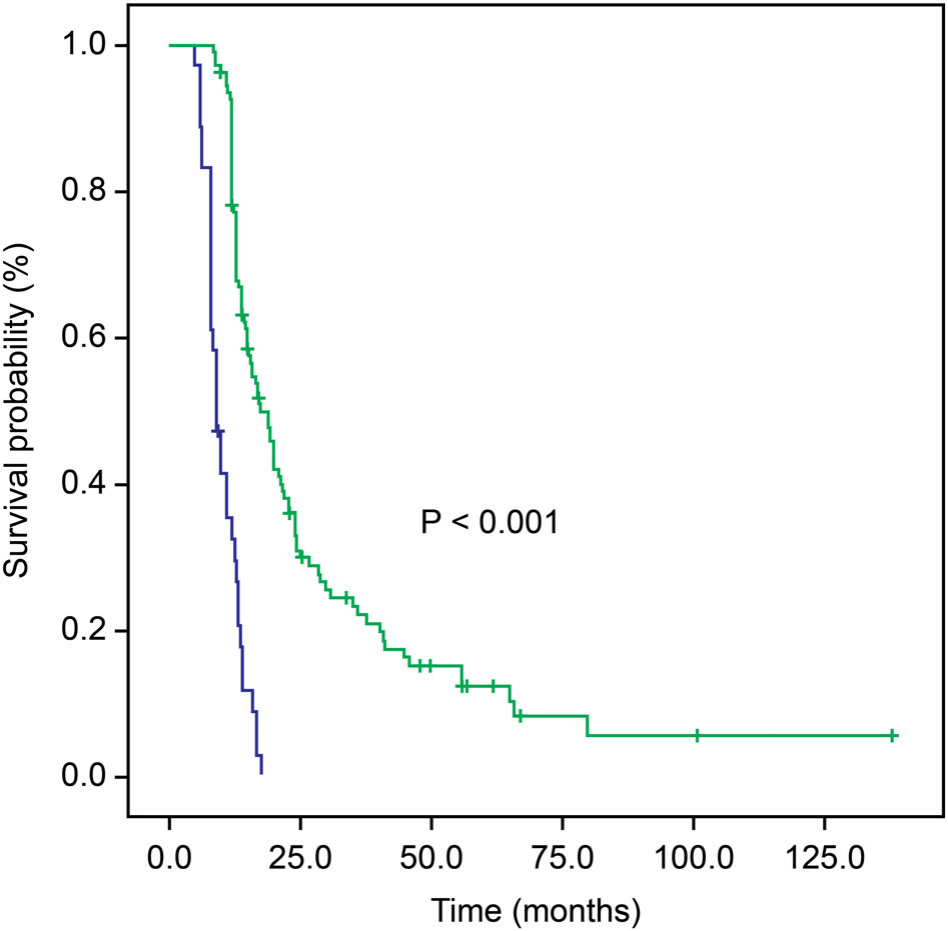
Kaplan‐Meier curves for overall survival (OS) according to the hemoglobin/red blood cell distribution width ratio (HRR) (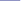) HRR < 0.985, (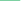) HRR ≥ 0.985, (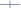) HRR < 0.985 censored, (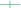) HRR ≥ 0.985 censored.

The median follow‐up time was 14 months (range, 5–138 months), and 140 patients died because of disease progression during the follow‐up. The average survival time of the entire cohort was 21 months. The one‐, two‐, and three‐year OS rates were 77%, 24%, and 13.7%, respectively, whereas the one‐, two‐, and three‐year PFS rates were 19.9%, 11.6%, and 6.8%, respectively. Patients were divided into high (≥0.985) and low (<0.985) HRR groups. Among all patients, 36 (24.7%) and 110 (75.3%) patients were in the low and high HRR group, respectively. The median OS in the whole group was 14 months (range, 5–138 months); the median OS in the low HRR group was nine months (95% confidence interval [CI], 7.5–10.4 months); and the median OS in the high HRR group was 17.5 months (95% CI, 14.6–20.4 months). The Kaplan‐Meier curve and log‐rank test showed significant differences between the high and low HRR groups (*P* < 0.001; Fig 1).

**Figure 2:**
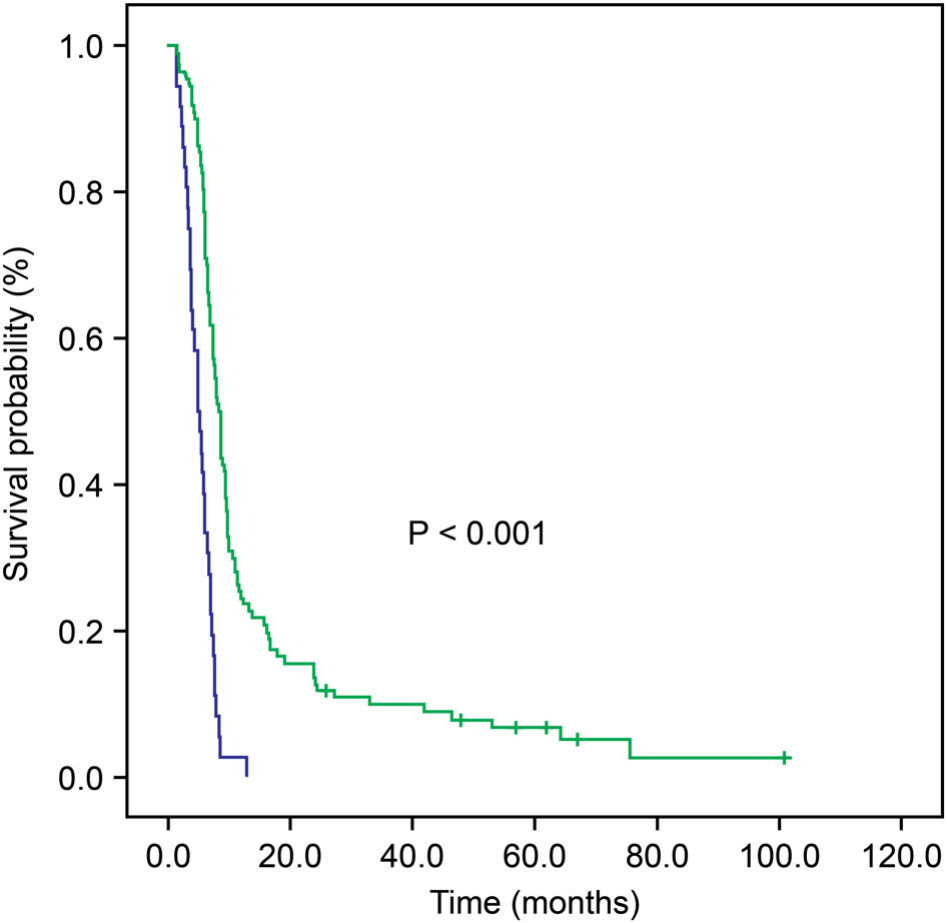
Kaplan‐Meier curves for progression‐free survival (PFS) according to the hemoglobin/red blood cell distribution width ratio (HRR) (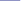) HRR < 0.985, (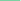) HRR ≥ 0.985, (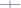) HRR < 0.985 censored, (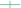) HRR ≥ 0.985 censored.

The median PFS was 7.5 months (range, 1.5–101 months) in the whole group, five months (95% CI, 3.8–6.2 months) in the low HRR group, and 8.5 months (95% CI, 7.6–9.4 months) in the high HRR group. Kaplan‐Meier curve and the log‐rank test showed significant differences between the high and low HRR groups (*P* < 0.001; Fig 2).

### Overall survival

In the univariate Cox proportional hazard regression analysis for OS, the following variables were found to be significantly associated with OS: ED stage (hazard ratio [HR], 2.137; 95% CI, 1.476–3.092; *P* < 0.001), first‐line etoposide combined with lobaplatin versus etoposide combined with cisplatin (HR, 1.662; 95% CI, 1.140–2.423; *P* = 0.008), first‐line sequential radiotherapy versus no radiotherapy (HR, 0.477; 95% CI, 0.320–0.710; *P* < 0.001), first‐line synchronous radiotherapy versus no radiotherapy (HR, 0.358; 95% CI, 0.216–0.593; *P* < 0.001), two‐cycle efficacy evaluation PD versus PR (HR, 2.535; 95% CI, 1.156–5.559; *P* = 0.02), high NLR (HR, 1.574; 95% CI, 1.079–2.294; *P* = 0.018), high PLR (HR, 1.464; 95% CI, 1.017–2.107; *P* = 0.04), high Hb (HR, 0.163; 95% CI, 0.094–0.283; *P* < 0.001), high RDW (HR, 4.000; 95% CI, 2.280–7.018; *P* < 0.001), and low HRR (HR, 5.449; 95% CI, 3.500–8.481; *P* < 0.001). Multivariate analysis was performed using factors that were found to be significant in the univariate analysis. The results showed that the following variables were significantly associated with OS: ED (HR, 1.691; 95% CI, 1.115–2.567; *P* = 0.014), first‐line etoposide combined with lobaplatin versus etoposide combined with cisplatin (HR, 1.725; 95% CI, 1.163–2.561; *P* = 0.007), first‐line sequential radiotherapy versus no radiotherapy (HR, 0.617; 95% CI, 0.401–0.948; *P* = 0.028), first‐line synchronous radiotherapy versus no radiotherapy (HR, 0.454; 95% CI, 0.266–0.774; *P* = 0.004), high Hb (HR, 0.394; 95% CI, 0.190–0.819; *P* = 0.013), and low HRR (HR, 3.782; 95% CI, 2.151–6.652; *P* < 0.001; Table 2).

**Table 2:** Univariate and multivariate statistical analyses of overall survival

	Univariate analysis			Multivariate analysis		
Variable	HR	95% CI	*P*‐value	HR	95% CI	*P*‐value
Sex						
Female	Reference					
Male	0.745	0.484–1.146	0.181			
Age						
≤60	Reference					
>60	1.389	0.973–1.982	0.071			
Smoking history						
No	Reference					
Yes	0.856	0.570–1.287	0.456			
Alcohol history						
No	Reference					
Yes	0.942	0.661–1.343	0.743			
Stage						
LD	Reference			Reference		
ED	2.137	1.476–3.092	<0.001	1.691	1.115–2.567	0.014
First‐line chemotherapy						
Etoposide + cisplatin	Reference			Reference		
Etoposide + lobaplatin	1.662	1.140–2.423	0.008	1.725	1.163–2.561	0.007
Etoposide + carboplatin	0.988	0.522–1.873	0.972	0.968	0.508–1.845	0.922
Combination with radiotherapy						
No	Reference			Reference		
Sequential radiotherapy	0.477	0.320–0.710	<0.001	0.617	0.401–0.948	0.028
Synchronous radiotherapy	0.358	0.216–0.593	<0.001	0.454	0.266–0.774	0.004
Two‐cycle efficacy evaluation						
PR	Reference			Reference		
SD	1.476	0.870–2.504	0.149	1.111	0.623–1.984	0.721
PD	2.535	1.156–5.559	0.02	1.704	0.712–4.077	0.232
NLR						
≤3	Reference			Reference		
>3	1.574	1.079–2.294	0.018	1.056	0.674–1.656	0.811
PLR						
≤165	Reference			Reference		
>165	1.464	1.017–2.107	0.04	0.845	0.545–1.311	0.452
Hb (g/dL)						
≤10.9	Reference			Reference		
>10.9	0.163	0.094–0.283	<0.001	0.394	0.190–0.819	0.013
RDW (%)						
≤14.1	Reference			Reference		
>14.1	4.000	2.280–7.018	<0.001	1.195	0.477–2.992	0.703
HRR						
≥0.985	Reference			Reference		
<0.985	5.449	3.5–8.481	<0.001	3.782	2.151–6.652	<0.001

*P*‐values <0.05 were statistically significant.

CI, confidence interval; ED, extensive disease; Hb, hemoglobin; HR, hazard ratio; HRR, hemoglobin/red blood cell distribution width ratio; LD, limited disease; NLR, neutrophil/lymphocyte ratio; PD, progressive disease; PLR, platelet/lymphocyte ratio; PR, partial response; RDW, red blood cell distribution width; SD, stable disease.

### Progression‐free survival

In the univariate Cox proportional hazard regression analysis for PFS, the following variables were found to be significantly associated with PFS: ED stage (HR, 1.939; 95% CI, 1.373–2.737; *P* < 0.001), first‐line etoposide combined with lobaplatin versus etoposide combined with cisplatin (HR, 1.455; 95% CI, 1.019–2.077; *P* = 0.039), first‐line sequential radiotherapy versus no radiotherapy (HR, 0.341; 95% CI, 0.231–0.504; *P* < 0.001), first‐line synchronous radiotherapy versus no radiotherapy (HR, 0.259; 95% CI, 0.159–0.420; *P* < 0.001), two‐cycle efficacy evaluation PD versus PR (HR, 76.021; 95% CI, 25.293–227.590; *P* < 0.001), high NLR (HR, 1.555; 95% CI, 1.089–2.220; *P* = 0.015), high PLR (HR, 1.488; 95% CI, 1.054–2.100; *P* = 0.024), high Hb (HR, 0.352, 95% CI, 0.230–0.541; *P* < 0.001), high RDW (HR, 2.786; 95% CI, 1.832–4.237; *P* < 0.001), and low HRR (HR, 3.562; 95% CI, 2.352–5.395; *P* = 0.001).

In the multivariate regression model, the following variables were found to be significantly associated with PFS: ED stage (HR, 1.777; 95% CI, 1.211–2.606; *P* = 0.003), first‐line sequential radiotherapy versus no radiotherapy (HR, 0.445; 95% CI, 0.289–0.685; *P* < 0.001), first‐line synchronous radiotherapy versus no radiotherapy (HR, 0.341; 95% CI, 0.206–0.565; *P* < 0.001), two‐cycle efficacy evaluation SD versus PR (HR, 1.838; 95% CI, 1.077–3.136; *P* = 0.026), two‐cycle efficacy evaluation PD versus PR (HR, 132.669; 95% CI, 33.739–521.685; *P* < 0.001), high RDW (HR, 1.817; 95% CI, 1.014–3.255; *P* = 0.045), and low HRR (HR, 2.112; 95% CI, 1.195–3.733; *P* = 0.01; Table 3).

**Table 3:** Univariate and multivariate statistical analyses of progression‐free survival

	Univariate analysis			Multivariate analysis		
Variable	HR	95% CI	*P*‐value	HR	95% CI	*P*‐value
Sex						
Female	Reference					
Male	0.731	0.489–1.094	0.128			
Age						
≤60	Reference					
>60	1.292	0.921–1.813	0.139			
Smoking history						
No	Reference					
Yes	0.821	0.559–1.205	0.314			
Alcohol history						
No	Reference					
Yes	0.955	0.681–1.339	0.789			
Stage						
LD	Reference			Reference		
ED	1.939	1.373–2.737	<0.001	1.777	1.211–2.606	0.003
First‐line chemotherapy						
Etoposide + cisplatin	Reference			Reference		
Etoposide + lobaplatin	1.455	1.019–2.077	0.039	1.224	0.840–1.785	0.292
Etoposide + carboplatin	1.231	0.683–2.219	0.49	1.067	0.561–2.030	0.843
Combination with radiotherapy						
No	Reference			Reference		
Sequential radiotherapy	0.341	0.231–0.504	<0.001	0.445	0.289–0.685	<0.001
Synchronous radiotherapy	0.259	0.159–0.420	<0.001	0.341	0.206–0.565	<0.001
Two‐cycle efficacy evaluation						
PR	Reference			Reference		
SD	1.481	0.887–2.474	0.134	1.838	1.077–3.136	0.026
PD	76.021	25.293–227.590	<0.001	132.669	33.739–521.685	<0.001
NLR						
≤3	Reference			Reference		
>3	1.555	1.089–2.220	0.015	1.477	0.998–2.184	0.051
PLR						
≤165	Reference			Reference		
>165	1.488	1.054–2.100	0.024	0.802	0.519–1.241	0.323
Hb (g/dL)						
≤12.3	Reference			Reference		
>12.3	0.352	0.230–0.541	<0.001	0.932	0.478–1.818	0.837
RDW (%)						
≤13.6	Reference			Reference		
>13.6	2.786	1.832–4.237	<0.001	1.817	1.104–3.255	0.045
HRR						
≥0.985	Reference			Reference		
<0.985	3.562	2.352–5.395	<0.001	2.112	1.195–3.733	0.01

*P*‐values <0.05 were statistically significant.

CI, confidence interval; ED, extensive disease; Hb, hemoglobin; HR, hazard ratio; HRR, hemoglobin/red blood cell distribution width ratio; LD, limited disease; NLR, neutrophil/lymphocyte ratio; PD, progressive disease; PLR, platelet/lymphocyte ratio; PR, partial response; RDW, red blood cell distribution width; SD, stable disease.

### Relationship between the Hb/RDW ratio and clinicopathological characteristics

We also examined the potential relationships between HRR and other clinicopathological features in SCLC patients. In the low HRR group, we found higher proportions of male patients (*P* = 0.001), patients with smoking history (*P* = 0.001), patients with ED (*P* = 0.03), and patients who had not undergone first‐line radiotherapy (*P* = 0.036). The patients in the low HRR group presented with higher NLR, higher PLR, higher Hb, and lower RDW values (all *P*‐values <0.001; Table 1).

## Discussion

In recent years, the prognosis of patients with NSCLC has greatly improved following progress in molecular biology techniques and the emergence of molecular targeted drugs and immunotherapy. However, because of the lack of both novel treatment regimens and simple and effective prognostic factors to evaluate prognosis, the survival of patients with SCLC has not significantly improved. Therefore, finding new and effective prognostic indicators is essential for improving the prognosis of patients with SCLC. Previous studies^24^, ^25^ have confirmed that HRR is associated with the prognosis of patients with ESCC, head and neck tumors, and other malignancies, but no relevant reports on patients with SCLC have been published. This study is the first to assess HRR in patients with SCLC, with the aim of evaluating the prognostic significance of baseline HRR in this population. The cutoff HRR value was determined using X‐tile software and used to categorize the study population into high and low HRR groups. The survival analysis showed that patients with a low HRR had a relatively poor OS and PFS, and the difference was statistically significant. Cox multivariate analysis revealed that the baseline HRR could be used as a predictor of survival in patients with SCLC.

Anemia is very common in patients with cancer, and it is associated with chronic blood loss,^14^ bone marrow infiltration,^31^ and inhibition of erythropoietin synthesis.^32^ The influence of anemia on the quality of life and prognosis of patients with cancer has recently attracted attention from researchers. Zhang *et al*. included 416 patients with stage I‐IV NSCLC, who were divided into normal and low Hb groups with or without anemia (Hb <12 g/dL for male patients, Hb <11 g/dL for female patients). That study confirmed that patients with NSCLC in the low Hb group had a poor prognosis.^33^ Pathak *et al*. evaluated 487 patients with stage I NSCLC treated with stereotactic body radiation therapy (SBRT) and reported that patients with anemia before treatment had increased local recurrence and distant metastasis rates; additionally, the OS rate of these patients was significantly lower.^34^

The potential diagnostic or prognostic significance of RDW in malignant tumors has also attracted increasing attention in recent years. Koma *et al*.^35^ retrospectively analyzed the data of 332 patients with lung cancer, including 109 women and 223 men who were divided into groups according to RDW values of ≥15% and <15%; the findings of their study confirmed that high levels of RDW are associated with advanced stage disease and indicate poor prognosis in the patients. Similarly, Ichinose *et al*.^22^ demonstrated that a high RDW value was significantly associated with increased morbidity and reduced survival in 992 elderly patients who had undergone NSCLC resection.

The present study showed that mortality risk in the high Hb value group was 0.394 times higher than that in the low Hb value group, which means that a low Hb level is a risk factor in terms of OS. There could be several reasons for the poor prognosis of patients with anemia. As Hb is the main molecule carrying oxygen in the body, a decrease in Hb concentration increases the number of hypoxic cells in the body, and the sensitivity of tumor cells to radiation and chemotherapy decreases under hypoxic conditions.^36^ Hypoxia can induce the formation of tumor cells with an invasive phenotype, leading to tumor progression and chemotherapy resistance.^37^ Moreover, hypoxia can also stimulate tumor neovascularization by activating multiple cell signaling pathways, thereby accelerating tumor invasion and metastasis.^38^, ^39^

However, no differences in PFS were observed between the two groups in this study, which could be because the cutoff Hb values are different for OS (10.9 g/dL) and PFS (12.3 g/dL). It is also possible that the small number of women enrolled had some influence on the findings. In this study, RDW was an independent prognostic factor for PFS in patients with SCLC (*P* = 0.045), and the risk of disease progression in the high RDW group was 1.817 times that in the low RDW group. The OS of the two groups was not statistically significant. These results may be related to the different cutoff values and the small number of participants. Although the mechanistic connection between RDW values and poor prognosis in cancer has not yet been established, there are several possible reasons for the poor prognosis. Inflammatory response, RDW, and several widely used plasma inflammatory markers, such as C‐reactive protein level, erythrocyte sedimentation rate,^17^ and interleukin‐6 level,^40^ are positively correlated with each other, and RDW is considered to be an inflammatory marker in patients with cancer. Additionally, malnutrition, which is associated with a deficiency of various minerals and vitamins,^41^, ^42^ such as iron, folic acid, and vitamin B12, as well as low protein levels,^22^ has also been linked to elevated RDW levels in patients with cancer. Finally, oxidative stress^43^ is related to red blood cell survival and could be a possible mechanism of action of RDW.

Although the relationship of Hb and RDW with the prognosis of patients with lung cancer has been confirmed, both parameters were influenced by diseases other than cancer. To minimize this influence, HRR may be a more reliable parameter to determine the prognosis of lung cancer. In the present study, univariate analyses of both PFS and OS identified Hb, RDW, and HRR as predictors of PFS and OS in patients with SCLC, but on multivariate analysis, HRR was found to be the only independent predictor of PFS and OS in patients with SCLC. Thus, compared with Hb and RDW, HRR has a more stable prognostic value. Furthermore, the cutoff value of HRR in this study (0.985) was very similar to that reported by Sun *et al*.^24^ in their study of patients with ESCC (0.989). The value is also very similar to the cutoff values for OS (1.019) and event‐free survival (EFS) (1.037) reported by Tristan *et al*.^25^ in head and neck tumors, as well as to the cutoff HRR value (0.88) reported by Yakup *et al*.^26^ in patients with NSCLC. Most importantly, the present study confirms that HRR can be used as an independent prognostic indicator in patients with SCLC. The risk of disease progression in the low HRR group was 2.112 times as that in the high HRR group (95% CI, 1.195–3.733; *P* = 0.01), and the risk of death was 3.782 times as that in the high HRR group (95% CI, 2.151–6.652; *P* < 0.001). Compared with other peripheral blood indicators that can predict the prognosis of SCLC, such as circulating tumor cells^8^ and micro‐RNA,^7^ HRR can be determined using routine CBC, which is inexpensive and easy to perform. Thus, the economic burden on patients is reduced to some extent, and clinical practice becomes more convenient.

This study has some limitations. First, as this was a retrospective study, it was impossible to exclude any potential conditions that affect Hb and RDW levels, such as autoimmune and systemic inflammatory diseases. Second, our study did not explore the relationships between posterior line therapy, adverse reactions, and HRR. Third, the patients in this study were from a single center, and the sample size was relatively small which might have led to selection bias. Finally, no uniform method was available for defining the cutoff HRR value due to the lack of standard methods for determining cutoff HRR values. In conclusion, this study was the first to explore the role of HRR in predicting the prognosis of patients with SCLC. Using a multivariate predictive model, HRR has been shown to be an independent predictor of OS and PFS, and HRR can therefore be used as a simple, feasible, and effective prognostic indicator in the clinical practice of SCLC therapy. Future multicenter, large‐sample, prospective studies are needed to validate the prognostic value of HRR.

## Disclosure

The authors have no conflicts of interest to declare.
